# Elastic Recovery Properties of Ultralight Carbon Nanotube/Carboxymethyl Cellulose Composites

**DOI:** 10.3390/ma14144059

**Published:** 2021-07-20

**Authors:** Kazuki Matsushima, Kenta Ono, Reo Yanagi, Naoto Shioura, Takahiro Segi, Tomonaga Ueno

**Affiliations:** Department of Chemical Systems Engineering, Graduate School of Engineering, Nagoya University, Furo-cho, Chikusa-ku, Nagoya 464-8603, Japan; matsushima.kazuki@e.mbox.nagoya-u.ac.jp (K.M.); ono.kenta@d.mbox.nagoya-u.ac.jp (K.O.); yanagi.reo@f.mbox.nagoya-u.ac.jp (R.Y.); shioura.naoto@j.mbox.nagoya-u.ac.jp (N.S.); segi.takahiro@h.mbox.nagoya-u.ac.jp (T.S.)

**Keywords:** ultralight materials, carbon nanotubes, composites, elastic recovery properties

## Abstract

Ultralight materials exhibit superelastic behavior depending on the selection, blending, and carbonization of the materials. Recently, ultimate low-density materials of 5 mg/cm^3^ or less have attracted attention for applications such as sensors, electrodes, and absorbing materials. In this study, we fabricated an ultralight material composed of single-walled carbon nanotubes (CNT) and sodium carboxymethyl cellulose (CMC), and we investigated the effect of density, composition, and weight average molecular weight of CMC on elastic recovery properties of ultralight CNT/CMC composites. Our results showed that the elastic recovery properties can be improved by reducing the density of the composite, lowering the mass ratio of CNTs, and using CMC with small molecular weight.

## 1. Introduction

Ultralight materials with excellent functionality are being researched for applications in fields such as resource and energy conservation, transportation, and aerospace [1,2,3,4,5,6,7,8]. In particular, ultralight materials based on nanocarbons, such as carbon nanotubes (CNTs) and graphene, have attracted attention in many applications such as sensors, electrodes, insulators, and adsorbents [9,10,11,12,13]. These ultralight materials are generally fabricated in the density range of less than 10 mg/cm^3^, and much research has been conducted on the correlation between their structure and mechanical properties [13,14,15,16,17,18,19]. In recent years, the weight of these ultralight materials has been further reduced, and materials of density approaching the air density (1.29 mg/cm^3^) are being developed [1,20]. In 2013, Sun et al. reported a graphene aerogel with a density of 0.16 mg/cm^3^ [21], which is approximately one sixth of air density and remarkably light. It has also been reported that ultralight aerogels with density less than air density can be levitated in air by controlling the temperature and selectively expanding the air inside the aerogel [22]. Such ultralight materials with ultimate lightness have the potential for further applications such as in communication and sky transportation [23,24].

There are limitations to enhance the mechanical strength of the materials that have density closer to air density. The low mechanical properties of these materials hinder their applications. Despite the constraint to improve the mechanical strength, the ultralight materials that exhibit elastic behavior are attracting attention in fields such as strain sensors [1,20,21]. Recently, materials with superelastic behavior and density of 5 mg/cm^3^ have been reported [1,20]. In particular, materials with a density of 1 mg/cm^3^ that recover 100% against 50% strain and materials with a density of 2.4 mg/cm^3^ that recover 100% against 99% strain by controlling their structure have been reported [1,21].

Such ultralight materials are fabricated by supercritical drying or freeze-drying of the solutions in which the materials are dispersed. This process controls pore structure of the material [25,26,27,28]. For example, in the freeze-drying process, structures are formed using ice as a template. The freeze-drying process has many advantages, including being inexpensive and environmentally friendly, and has been used in many studies [29,30]. The structure of the ultralight materials is generally controlled by ice growth during the freeze-drying process; hence, it is important to control the ice growth to control the structure precisely. In the freezing process of dispersed solutions, ice growth is significantly affected by the concentration and molecular weight of the solute [31]. To improve the recovery properties of the low-density porous materials, it is necessary to control the pore structure to a high degree, which is done by controlling the ice growth during the freeze-drying process. However, there are only limited studies available on the effects of solute concentration and molecular weight on these properties.

In this study, we manufactured the ultralight materials with a density less than 5 mg/cm^3^ by compositing single-walled CNT and sodium carboxymethyl cellulose (CMC) using freeze-drying. Single-walled CNTs facilitate the fabrication of materials in the density range approaching air density due to their high aspect ratio and mechanical properties [22]. CMC was used as dispersant [32,33]. Since CMC have OH groups in their structure, they can form hydrogen bonds with the oxygen-containing functional groups present at the ends of CNTs, thus allowing CNTs to be well dispersed in water. In order to improve the elastic properties of these composite materials, we investigated the effects of material density, composition, and molecular weight of water-soluble polymers. As a result, the elastic recovery properties of the CNT/CMC composites were improved by reducing the density, lowering the CNT mass ratio, and using CMC with small molecular weight.

## 2. Materials and Methods

### 2.1. Materials

CNT (eDIPS EC 2.0, Meijo Nano Carbon Co., Ltd., Aichi, Japan) with a diameter of 1–3 nm and a length of 1 µm or more were used to fabricate the composite material. The transmission electron microscopy (TEM) image and Raman spectrum of CNT are shown in Figure S1 of the Supplementary Information (SI). Four types of sodium carboxymethyl cellulose (CMC) with different molecular weights were used. CMC with weight average molecular weights of 100,000, 250,000, 660,000, and 820,000 were labeled as CMC1, CMC2, CMC3 and CMC4, respectively. The properties (degree of etherification, viscosity, molecular weight) of CMC are shown in Table S1 of Supplementary Materials.

### 2.2. Fabrication of Ultralight Materials

**Figure 1:**
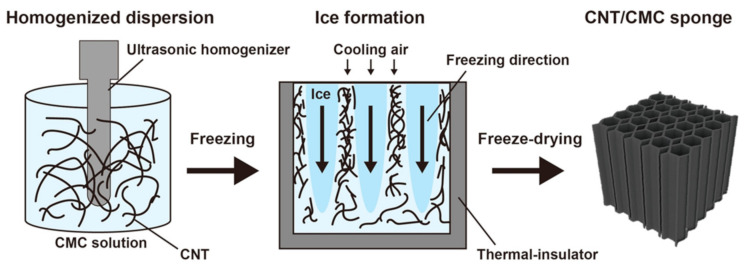
Schematic illustration of the preparation of a CNT/CMC sponge.

Figure 1 schematically illustrates the preparation of a CNT/CMC sponge. CNT and CMC components were added to distilled water. The samples compositions were adjusted to 1.3, 2.5, and 5.0 g/L to fabricate composites with apparent densities of 1.3, 2.5, and 5.0 mg/cm^3^, respectively. The CNT mass ratios were 0, 10, and 20%, respectively. The dispersions were ultrasonically agitated for 12 min using an ultrasonic homogenizer (Sonifier 450, Emerson Japan Ltd., Tokyo, Japan), These dispersions were cooled at 5 °C for 30 min in a low-temperature water bath and were frozen at −80 °C for 3 h in a small ultra-low temperature bath (VT-78, Nippon Freezer Co., Ltd. Tokyo, Japan). The dispersions were uniaxially frozen by covering the sides and bottom with a thermal-insulating material. The ice that grows in the sample during freezing forms the pore structure of the sample after fabrication. Finally, the dispersions were freeze-dried (FDU-12AS freeze dryer, As One Corporation, Osaka, Japan). From the above process, low-density CNT/CMC sponge composite materials were fabricated. The dimensions of CNT/CMC sponge were 40 mm × 40 mm × 40 mm each. According to the density and composition of each sample, it is marked CNT/CMCX (density_CNT mass ratio), where X = 1–4 according to CMC molecular weight. For example, CNT/CMC1(1.3_10) means the sample using CMC1 with a density of 1.3 mg/cm^3^ and a CNT mass ratio of 10%.

### 2.3. Analysis Method

We examined the surface and the cross-sections of the samples using field-emission scanning electron microscopy (FE-SEM) (S4800, Hitachi High-Technologies Corporation, Tokyo, Japan) and 3D X-ray microscopy (X-ray CT) (SKYSCAN1275, Bruker Corporation, Billerica, MA, USA). The mechanical properties were measured through compression tests. The elastic modulus at 20% strain and the recovery rate when compressed to 80% strain were measured from the obtained stress–strain curves. The crosshead speed during the test was 1 mm/min. All the tests were conducted under room temperature and dried condition. The pore volume, Brunauer-Emmett-Teller (BET) surface area, and pore-size distribution of the samples were obtained by using automatic specific surface area/fine pore distribution measurement device (TriStar Ⅱ 3020, Micromeritics Instrument Corporation, Norcross, GA, USA).

## 3. Results

**Figure 2:**
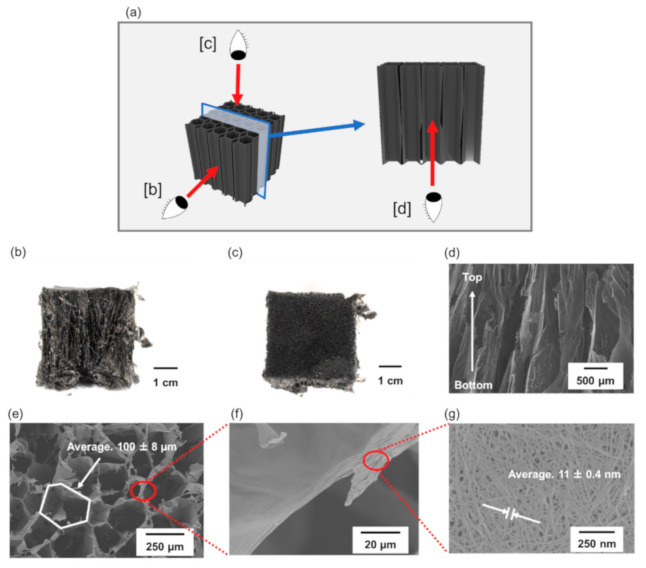
(**a**) Schematic of CNT/CMC2(1.3_10) sample observations, [b], [c] and [d] represent the directions in which (**b**), (**c**) and (**d**) are observed. (**b**) Photograph of the side. (**c**) Photograph of the top surface. (**d**) SEM image of the cross-section. (**e**) SEM image of the top surface. (**f**) SEM image of the sheet structure forming the skeleton. (**g**) SEM image of the sheet surface in magnification.

Figure 2 shows photographs and SEM images of CNT/CMC2(1.3_10). Figure 2b,c show photographs of the side and top parts of the sample, respectively. Based on the images, we could suggest that the material was fabricated without shrinkage during the freeze-drying process, despite its low density of 1.3 mg/cm^3^. When multi-walled CNTs were used, they cracked even when the density was set to 5.0 mg/cm^3^ (Figure S2 of Supplementary Materials). Figure 2d,e show SEM images of the cross-section and top part of the sample, respectively. Figure 2f shows an SEM image of the skeleton, which forms a honeycomb structure, and Figure 2g shows the skeleton surface in magnitude. Figure 2d,e show that the sponge has honeycomb-shaped pores oriented from the top to bottom attributed to the uniaxial freezing. Furthermore, the oriented structure was also observed by X-ray CT imaging shown in Figure S3 of Supplementary Materials. We calculated pore size taking into account the average radius of the pores, which was calculated by approximating the honeycomb cells with a circle. The pore size was calculated at 100 ± 8 μm. The SEM images of CNT/CMC2(2.5_10) and CNT/CMC2(5_10) are show in Figure S4 and Figure S5 of Supplementary Materials. Furthermore, from the SEM image of Figure 2g, we calculated that the mean diameter of the CNT fiber bundles was 11 ± 0.4 nm. Honeycomb-shaped pores were formed at the top of the CNT/CMC sponge. This is due to the cooling and freezing process starting from the top part of the dispersion by cooling air. First, hexagonal nuclei of water were grown uniformly at the top part of the dispersion by the cooling air. The nuclei were grown in the axial and radial directions and then formed a hexagonal cylinder. Honeycomb-shaped pores were formed by the removal of ice crystals during freeze-drying.

**Figure 3:**
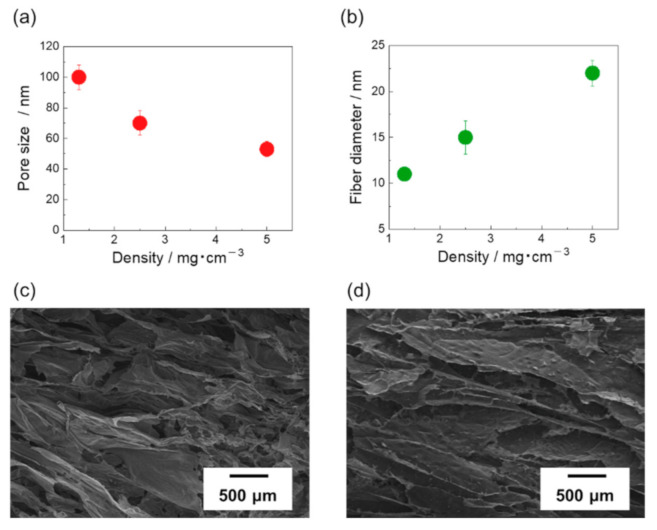
(**a**) Pore size versus density. (**b**) Fiber diameter versus density. (**c**) SEM images of the wall in CNT/CMC2(1.3_10) sponge. (**d**) SEM images of the wall in CNT/CMC2(5_10) sponge.

Figure 3a shows the relationship between the CNT/CMC2 sponge density and pores size. The average pore sizes were calculated at 100 ± 8, 70 ± 8, and 54 ± 5 μm for densities of 1.3, 2.5, and 5 mg/cm^3^, respectively. The pore size decreased as the density increased (Figure S4 of Supplementary Materials). Figure 3b shows the relationship between the density of the CNT/CMC2 sponge and the mean diameter of the CNT fiber bundles in the CNT/CMC skeleton. At a density of 1.3, 2.5, and 5 mg/cm^3^, the mean diameters of CNT fiber bundles were calculated at 11 ± 0.4 nm, 15 ± 1.8 nm, and 22 ± 4 nm, respectively. As the density increased, the mean diameter of CNT fiber bundles increased (Figure S5 of Supplementary Materials). Figure 3c,d show SEM images of the CNT/CMC2(1.3_10) and CNT/CMC2(5_10) walls, respectively. The folds and wrinkles in the inner wall increase with a decrease in density. The higher the density, the higher the concentration of the solute present in the dispersion. Consequently, more ice nuclei were formed, which resulted in a finer structure and smaller pore size. In addition, the higher the solute concentration, the more the freezing point depression. Hence, the supercooling degree was higher and the ice nucleation was easier, leading to a smaller pore size. When the density was decreased, the concentration of CNT in the dispersion liquid decreased. Therefore, when the dispersing material was dispersed at the same composition ratio, the dispersion became easier, and the diameter of the CNT fiber bundles became smaller.

**Figure 4:**
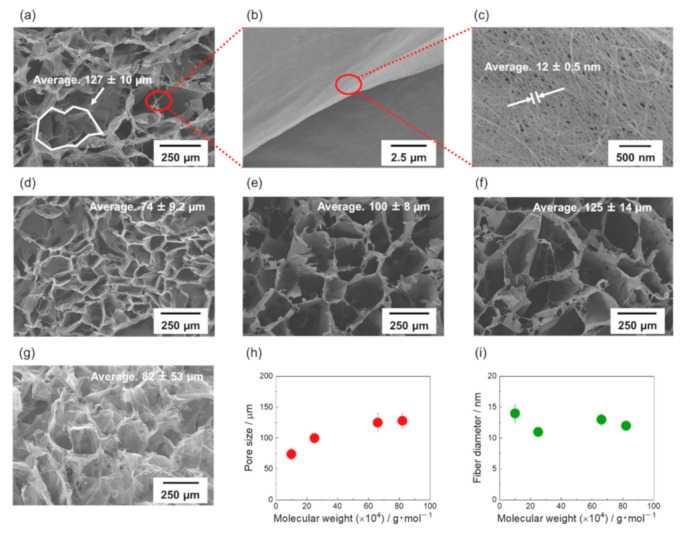
(**a**) SEM image of top surface of CNT/CMC4(1.3_10). (**b**) SEM image of the sheet structure forming the skeleton of CNT/CMC4(1.3_10). (**c**) SEM image of the sheet surface of CNT/CMC4(1.3_10) in magnitude. (**d**) SEM image of top surface of CNT/CMC1(1.3_10). (**e**) SEM image of top surface of CNT/CMC2(1.3_10). (**f**) SEM image of top surface of CNT/CMC3(1.3_10). (**g**) SEM image of top surface of CNT/CMC1(1.3_20). (**h**) Relationship between weight average molecular weight of CMC and average pore size and (**i**) relationship between weight average molecular weight and average diameter of CNT fiber bundles in CNT/CMC sheets.

Figure 4a–c show SEM images of the CNT/CMC4(1.3_10). Figure 4a shows SEM images of the top view. Figure 4b shows the SEM image of the skeleton. From Figure 4a, it was observed that CNT/CMC4(1.3_10) had a honeycomb structure as well, similar to that of CNT/CMC2(1.3_10) (Figure 2e). From Figure 4c, the average diameter of CNT fiber bundles was calculated at 12 ± 0.5 nm. The SEM images of CNT/CMC1(1.3_10), CNT/CMC2(1.3_10), CNT/CMC3(1.3_10) and CNT/CMC1(1.3_20) are show in Figure 4d–g. Figure 4h shows the relationship between the weight average molecular weight of CMC and the average pore size. The pore size increased with an increase in the molecular weight. As the molecular weight of the CMC decreases, the molar-mass concentration of the dispersion increases, and the freezing point depression becomes more pronounced. Since the freezing point depression facilitates nucleation, the pore size of the CNT/CMC sponge with a small molecular weight CMC became smaller. Figure 4i shows the relationship between the weight average molecular weight of CMC and the average diameter of CNT fiber bundles in CNT/CMC skeleton. A slight decrease in diameter was observed when the molecular weight of CMC increased (Figure S6 of Supplementary Materials). Table S2 shows the pore volume and BET surface area of the samples, and Figure S7 shows the pore-size distribution of the samples.

**Figure 5:**
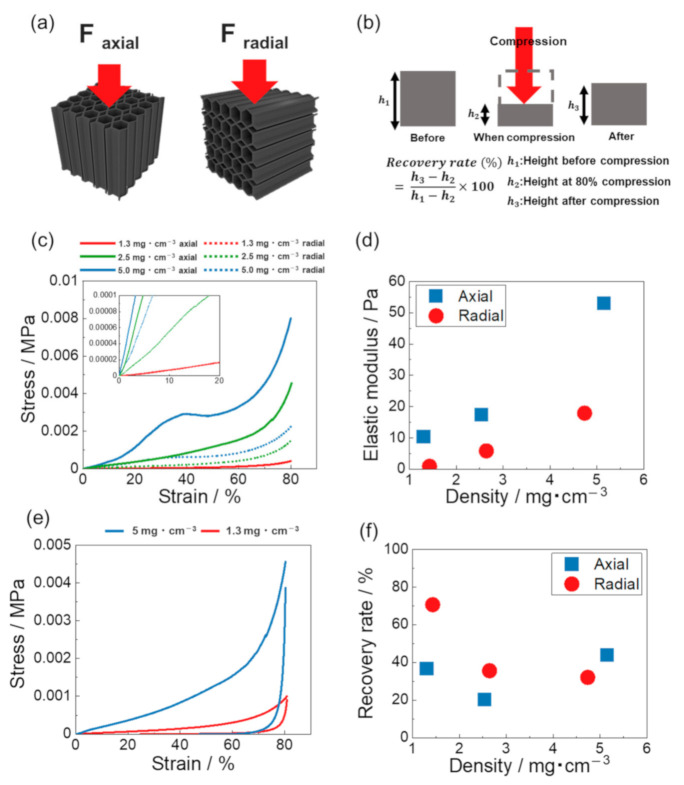
(**a**) Schematic of the compression directions. (**b**) Recovery rate calculation method. (**c**) Stress–strain curves of CNT/CMC2 (1.3_10), CNT/CMC2 (2.5_10), and CNT/CMC2 (5_10) in different compression directions. (**d**) Relationship between density and elastic modulus. (**e**) Stress–strain curves in radial direction for CNT/CMC2 (1.3_10) and CNT/CMC2 (5_10). (**f**) Relationship between density and recovery rate of the samples in different compression directions. (In (**d**,**f**), x-axis shows the actual measured density).

Figure 5a shows a schematic of the direction of the compression tests. Because the CNT/CMC sponge is an anisotropic material, compression tests were performed in two directions, parallel and perpendicular to the orientation of the material with respect to the orientation direction (axial and radial compression, respectively). The recovery rate was calculated by the ratio of the recovered height to the compressed distance as shown in Figure 5b and Equation (1):

(1) $$Recovery\;rate\left(\%\right)=\frac{h_{3}-h_{2}}{h_{1}-h_{2}}\times 100,$$

where, *h*_1_ is the height before compression, *h*_2_ is the height at 80% compression, and *h*_3_ is the height after compression. Figure 5c shows the stress–strain curves obtained from compression tests in two different directions for the samples with different densities. At densities of 2.5 and 5 mg/cm^3^, the slopes of the stress–strain curves for up to 20% strain were larger in the axial than in radial compression. On the other hand, there was almost no difference in the stress–strain curves at 1.3 mg/cm^3^. Yield points were observed only in the axial compression. At 1.3 mg/cm^3^ the yield point is not clearly discernible, but at 2.5 mg/cm^3^ the yield point is at strain of 30%, stress of 0.0006 MPa, and at 5.0 mg/cm^3^ the yield point is at strain of 40%, stress of 0.003 MPa (Figure S8a–c). The honeycomb-structured skeleton was oriented in the axial direction, when a load was applied in the axial direction, the central part of the honeycomb wall buckled and plastic deformation occurred. As a result, a yield point appeared on the stress–strain curve. On the other hand, in the radial compression, the stress is applied to the corners of the honeycomb structure. The honeycombs begin to deform and the pores are crushed before the buckling of the honeycombs. Therefore, no yield point appeared. Figure 5d shows the relationship between the density and elastic modulus calculated from the stress–strain curves of Figure 5c. The elastic modulus of CNT/CMC sponge decreased with decreasing density regardless of the compression direction. The elastic modulus of the axial compression is higher than that of radial compression in any density range. Figure 5e shows the stress–strain curves of CNT/CMC2 sponges with densities of 1.3 and 5 mg/cm^3^ when compressed radially. At a density of 5 mg/cm^3^, the compressive stress at 80% strain was higher than that of 1.3 mg/cm^3^, and the recovery rate was calculated at 38%. On the other hand, at 1.3 mg/cm^3^, the recovery rate was improved to 74%. Figure 5f shows the relationship between the density and the recovery rate of the samples under axial and radial compression. In axial compression, the recovery rate was approximately 40% for all densities, whereas in radial the recovery rate increased with decreasing density. As the density decreased, the number of folds and wrinkles in the inner wall increased (Figure 3c,d). Figure S9 shows the schematic diagrams of CNT/CMC sponges with different densities under compression. The decrease in the density not only allowed the walls to deform more elastically but also reduced the adhesive force by reducing the contact points between the walls [34]. As a result, the recovery rate improved.

**Figure 6:**
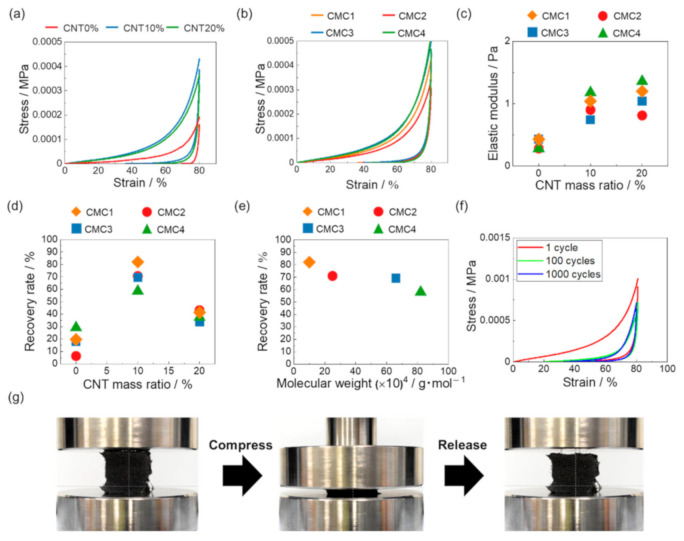
(**a**) Stress–strain curves of CNT/CMC2(1.3_X) with different CNT mass ratios (X = 0, 10, 20%). (**b**) Stress–strain curves of CNT/CMCX(1.3_10) with different CMC molecular weights (X = 1–4). (**c**) Relationship between CNT mass ratio and elastic modulus of the samples with different CMC molecular weights. (**d**) Relationship between CNT mass ratio and recovery rate of the samples with different CMC molecular weights. (**e**) Relationship between CMC molecular weight and recovery rate of CNT/CMCX(1.3_10). (**f**) Stress–strain curve after the cycle compression test for CNT/CMC1(1.3_10). (**g**) Photographs during the recovery test of CNT/CMC1(1.3_10).

Figure 6a shows the stress–strain curves of CNT/CMC2 sponge with 1.3 mg/cm^3^ and different CNT mass ratio. At 0% CNT mass ratio, the compressive stress at 80% strain was small. However, the increase in CNT concentration improved the compressive stress. The compressive stress at 80% strain remained almost stable for 10% and 20% CNT mass ratios. Figure 6b shows the stress–strain curves of CNT/CMC(1.3_10) for various weight average molecular weights of CMC. The compressive stress at 80% strain remained stable with different molecular weights. Figure 6c shows the relationship between the CNT mass ratio and the elastic modulus of CNT/CMC sponges with different molecular weights of CMC. The elastic modulus tended to increase with increasing CNT mass ratio. Then, the change in the molecular weight did not considerably affect the elastic modulus. Figure S10 shows relationship between CMC molecular weight and maximum stress when the CNT mass ratio is 10%. As the molecular weight increases, the maximum stress tends to increase. Figure 6d shows the relationship between the CNT mass ratio and the recovery rate under different weight average molecular weights. The recovery rate of 10% CNT and 20% CNT was higher than that of 0% CNT, confirming that the recovery rate was improved by adding CNT. This is because the addition of CNT increased the strength of the wall and made it more elastic. Then, comparing 10% CNT and 20% CNT, 10% CNT showed a higher recovery rate. This is because the integrity of the honeycomb structure was maintained when the CNT ratio was lower (Figure 4d,g). This clean honeycomb structure allowed a large amount of strain energy to be stored, resulting in a higher recovery rate. In this study, the optimal CNT mass ratio was 10%. Figure 6e shows the relationship between the CMC molecular weight and the recovery rate when the CNT mass ratio was 10%. When the molecular weight of CMC was 820,000 (CMC4), the recovery rate was 58%, but when the molecular weight was 100,000 (CMC1), it was 82%, indicating that the recovery rate increased when the molecular weight of CMC decreased. As shown in Figure 4h, as the CMC molecular weight decreased, the pore size of the CNT/CMC sponge decreased. The smaller the pore size, the more stress is distributed during compression. Therefore, the CNT/CMC sponge with a small molecular weight CMC was less likely to buckle and showed a higher recovery rate. Figure 6f shows the stress–strain curve after the cycle compression test for CNT/CMC1(1.3_10), and Figure S11 shows the recovery rate after the test. Figure 6g shows the photograph of the sample during the recovery test. As it can be seen, the compression can be performed without any internal deformation during the test, and the recovery is instantaneous after unloading (see Video S1 in Supplementary Materials). 

## 4. Conclusions

In this study, we fabricated an ultralight material composed of single-walled CNT and CMC, and we investigated the effect of density, composition, and weight average molecular weight of CMC on composite properties. When the CNT/CMC sponge was compressed in the radial direction, it showed a recovery strength, and the recovery rate improved as the density decreased. In addition, the recovery rate of the CNT/CMC sponge was improved by lowering the CNT mass ratio and using CMC with small molecular weight. Although further improvement of the recovery rate and how long the function can be maintained is still an issue, it is expected to be applied to sound absorbing materials, electromagnetic wave shielding materials, and materials that float in the air.

## Data Availability

The data presented in this study are available on request from the corresponding author.

## Supplementary Materials

The following are available online at https://www.mdpi.com/article/10.3390/ma14144059/s1. Figure S1: (a) Photograph, (b) TEM image, and (c) Raman spectrum of CNT used in this study. Table S1: The degrees of etherification and weight average molecular weights of the CMC used in this study. Figure S2: Sample appearance when the materials used were changed (a) CNT/CMC2(5_20) and (b) MWCNT/CMC2(5_20). Figure S3: (a) X-ray CT image of a CNT/CMC sponge fabricated by normal frozen process. (b) X-ray CT image of a CNT/CMC sponge fabricated by uniaxially frozen process. (c) Cross-sectional views of the CNT/CMC sponge fabricated by normal frozen process. (d) Cross-sectional views of the CNT/CMC sponge fabricated by uniaxially frozen process. (e) Graphs of the orientation distribution of the CNT/CMC sheet in CNT/CMC sponge fabricated by normal frozen process. (f) Graphs of the orientation distribution of the CNT/CMC sheet in CNT/CMC sponge fabricated by uniaxially frozen process. Figure S4: SEM images of the top parts ((a), (c)) and sheets surfaces ((b), (d)) of the CNT/CMC2(2.5_10) ((a), (b)) and CNT/CMC2(5_10) ((c), (d)). Figure S5: SEM images of the cross-sections ((a), (c)) and sheets surfaces ((b), (d)) of the CNT/CMC2(2.5_10) ((a), (b)) and CNT/CMC2(5_10) ((c), (d)). Figure S6: SEM images of the CNT/CMC sheets, and CNT/CMC sheet surfaces with different weight average molecular weight; (a) and (c) CNT/CMC1(1.3_10); (b) and (d) CNT/CMC3(1.3_10). Table S2: Pore volume and BET surface area obtained from BET N_2_ sorption measurement. Figure S7: The pore-size distribution of the samples; (a): CNT/CMC1(1.3_10), (b): CNT/CMC2(1.3_10), (c): CNT/CMC3(1.3_10), (d): CNT/CMC4(1.3_10), (e): CNT/CMC1(1.3_20). Figure S8: (a), (b), (c) Stress–strain curves for the axial compression; (a) CNT/CMC2 (1.3_10), (b) CNT/CMC2(2.5_10), (c) CNT/CMC2(5_10). (d), (e), (f) Stress–strain curves for radial compression; (d) CNT/CMC2(1.3_10), (e) CNT/CMC2(2.5_10), (f) CNT/CMC2(5_10). Figure S9: Schematic diagrams of CNT/CMC sponge under compression; (a) CNT/CMC2(1.3_10), (b) CNT/CMC2(5_10). Figure S10: Relationship between CMC molecular weight and maximum stress when the CNT mass ratio was 10%. Figure S11: Relationship between cycle number and recovery rate when repeatedly compressing CNT/CMC1(1.3_10). Video S1: supplementary movie of the compressed CNT/CMC1(1.3_10).
