# Supplementary material for: Elastic Recovery Properties of Ultralight Carbon Nanotube/Carboxymethyl Cellulose Composites
Source: Materials (Basel). 2021 Jul 20;14(14):4059. doi: 10.3390/ma14144059 (PMC8306045; doi:10.3390/ma14144059)
Supplement: Supplementary file 1 [file materials-14-04059-s001.zip › materials-1288930-supplementary-for pub.pdf]

materials

MDPI

Supplementary Materials

Elastic Recovery Properties of Ultralight Carbon Nanotube/ Carboxymethyl Cellulose Composites

Kazuki Matsushima, Kenta Ono, Reo Yanagi, Naoto Shioura, Takahiro Segi and Tomonaga Ueno *

Department of Chemical Systems Engineering, Graduate School of Engineering, Nagoya University, Furo-cho, Chikusa-ku, Nagoya 464-8603, Japan; matsushima.kazuki@e.mbox.nagoya-u.ac.jp (K.M.); ono.kenta@d.mbox.nagoya-u.ac.jp (K.O.); yanagi.reo@f.mbox.nagoya-u.ac.jp (R.Y.); shioura.naoto@j.mbox.nagoya-u.ac.jp (N.S.); segi.takahiro@h.mbox.nagoya-u.ac.jp (T.S.)

* Correspondence: ueno.tomonaga@material.nagoya-u.ac.jp

In Figure S1(a), (b), and (c) the photograph, TEM image, and Raman spectrum of the CNT are shown, respectively. From Figure S1 (b), the diameter of the CNT was observed at 1-3 nm. The purity was over 90%. Furthermore, from the Raman spectrum of Figure S1 (c), the G band peak at 1590 cm^{-1} was observed. No D band peak usually located around 1300 cm^{-1}, was observed. These results indicate that the CNT used, had high purity and crystallinity. Although it is difficult to determine the exact length of the CNT, according to TEM analysis the length was estimated at more than 1 μm.

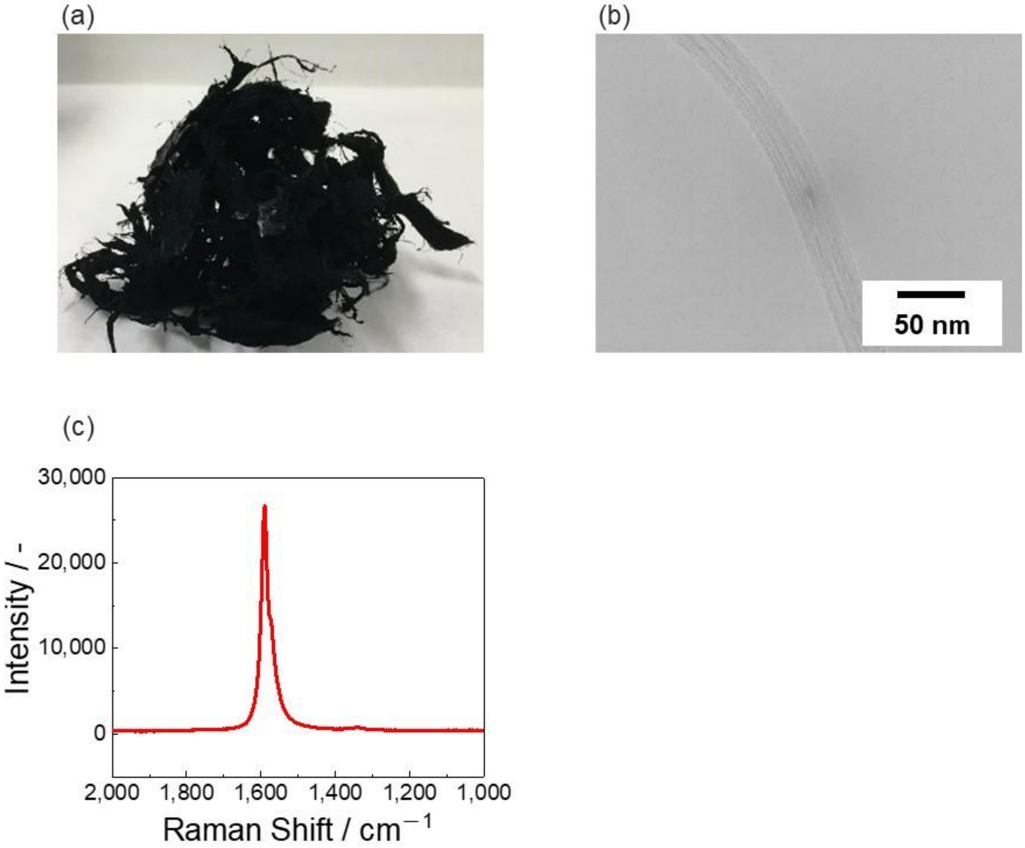

Figure S1. (**a**) Photograph, (**b**) TEM image, and (**c**) Raman spectrum of CNT used in this study.

Table S1 shows the properties (degree of etherification and weight average molecular weight) of the CMC used in this study.

Table S1. The degrees of etherification and weight average molecular weights of the CMC used in this study.

Sample Name	Manufacturer	Product Name	Degree of Etherification	Weight Average Molecular Weight (g/mol)
CMC1	Fujifilm Wako Pure Chemical Co., Ltd	-	0.65-0.85	100,000
CMC2	Daicel Finechem Co., Ltd	CMC1120	0.6-0.8	250,000
CMC3	Daicel Finechem Co., Ltd	CMC1160	0.6-0.8	660,000
CMC4	Daicel Finechem Co., Ltd	CMC1190	0.6-0.8	820,000

Figure S2 shows the appearance of the samples when the materials used were changed. Sample with a density of 5 mg/cm^3 was fabricated using Multi-walled Carbon Nanotubes (MWCNTs) (150 nm diameter) instead of SWCNTs (1-3 nm diameter), and the sample cracked immediately after fabrication (Figure S2 (b)).

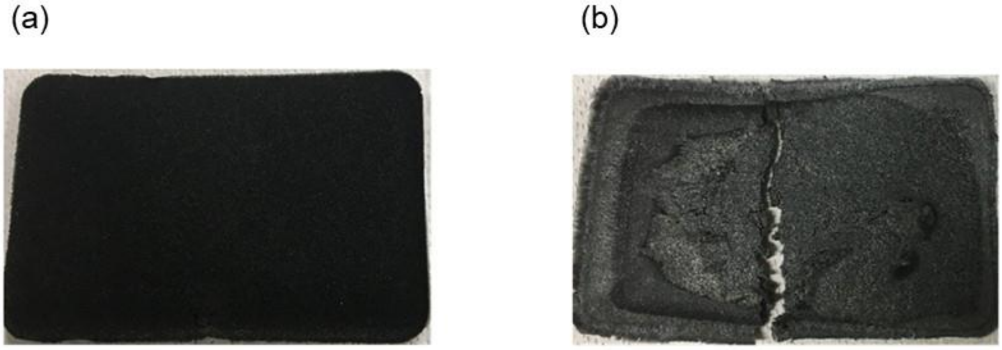

Figure S2. Sample appearance when the materials used were changed (a) CNT/CMC2(5_20) and (b) MWCNT/CMC2(5_20).

Figures S3 (a) and (b) show the X-ray CT images of CNT/CMC composite sponges, and (c) and (d) correspond to cross-sectional views of CNT/CMC composite sponges. Figure S3 (e) and (f) show the distributions of the angle with respect to Y axis obtained by image analysis of cross-sectional views. The CNT/CMC composite sponges used in the X-ray CT scan had Pt nanoparticles added to increase the sensitivity of detection. Figures S3 (a), (c), and (e) show the results of the CNT/CMC composite sponge fabricated by normal frozen process and Figure S3 (b), (d), and (f) show the results of CNT/CMC composite sponge fabricated by uniaxially frozen process used in this study. The density of these CNT/CMC composite sponge were 5 mg/cm^3. From Figure S3 (a), (c), and (e), the non-orientated structure was observed. On the other hand, from Figure S3 (b), (d), and (f), Z-axis aligned structure was observed.

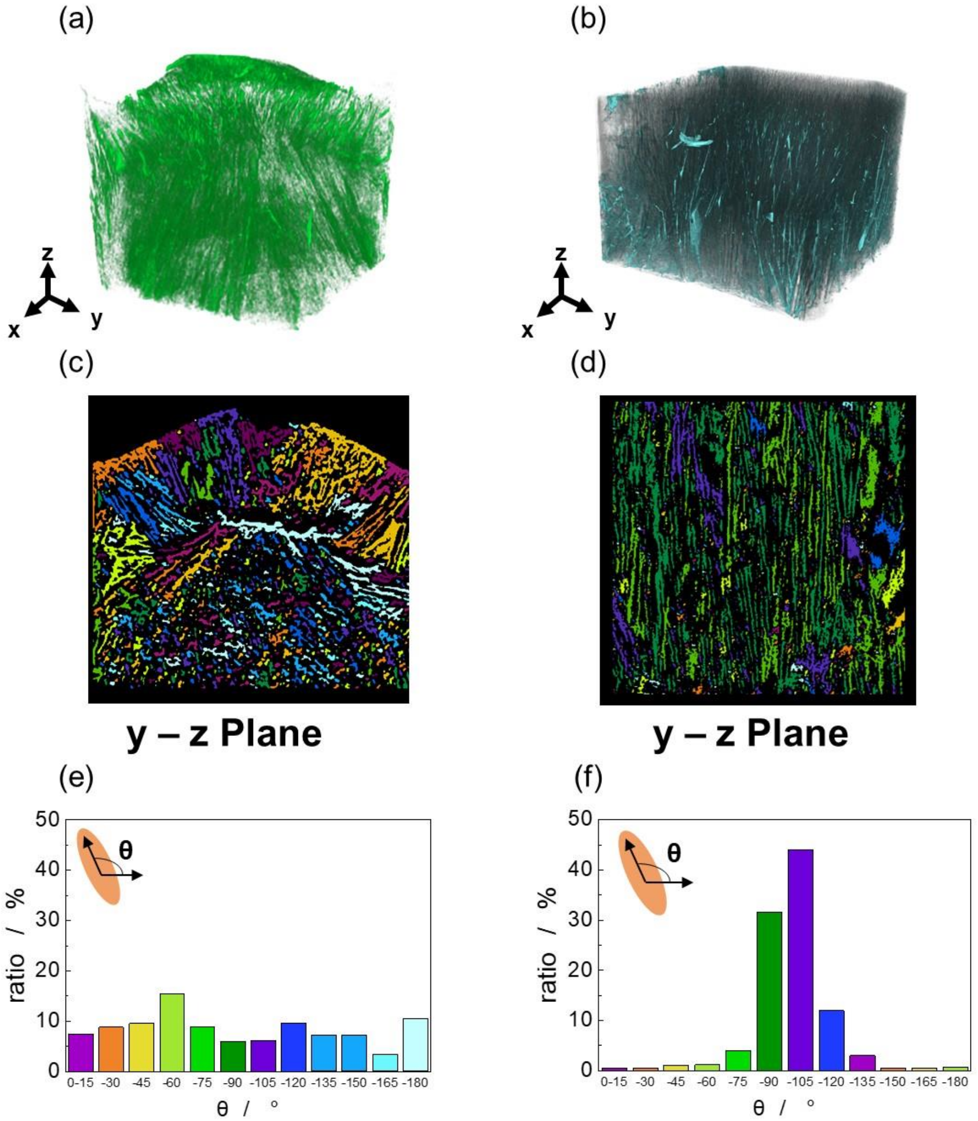

Figure S3. (**a**) X-ray CT image of a CNT/CMC sponge fabricated by normal frozen process. (**b**) X-ray CT image of a CNT/CMC sponge fabricated by uniaxially frozen process. (**c**) Cross-sectional views of the CNT/CMC sponge fabricated by normal frozen process. (**d**) Cross-sectional views of the CNT/CMC sponge fabricated by uniaxially frozen process. (**e**) Graphs of the orientation distribution of the CNT/CMC sheet in CNT/CMC sponge fabricated by normal frozen process. (**f**) Graphs of the orientation distribution of the CNT/CMC sheet in CNT/CMC sponge fabricated by uniaxially frozen process.

Figure S4 shows the SEM images of the top parts and sheets surfaces of the CNT/CMC2(2.5_10) ((a), (b)) and CNT/CMC2(5_10) ((c), (d)). The CNT/CMC sheets were composed of CNT and CMC and formed a honeycomb-shaped porous structure.

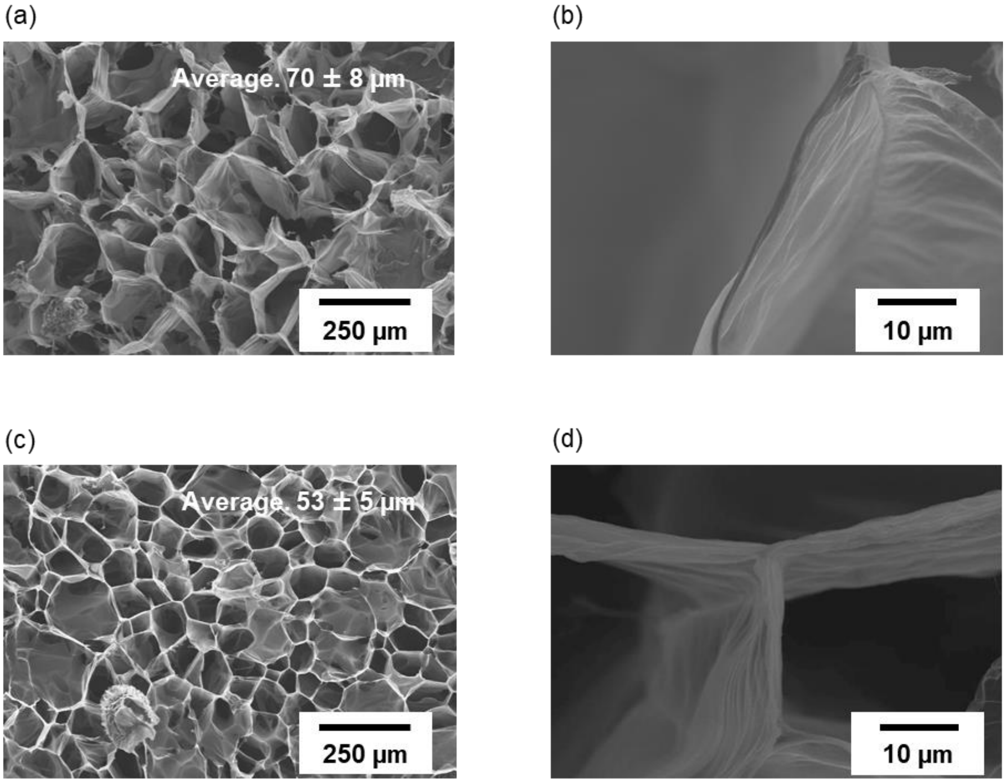

Figure S4. SEM images of the top parts ((**a**), (**c**)) and sheets surfaces ((**b**), (**d**)) of the CNT/CMC2(2.5_10) ((a), (b)) and CNT/CMC2(5_10) ((**c**), (**d**)).

Figure S5 shows the SEM images of the cross-sections and sheets surfaces of the CNT/CMC2(2.5_10) ((a), (b)) and CNT/CMC2(5_10) ((c), (d)).

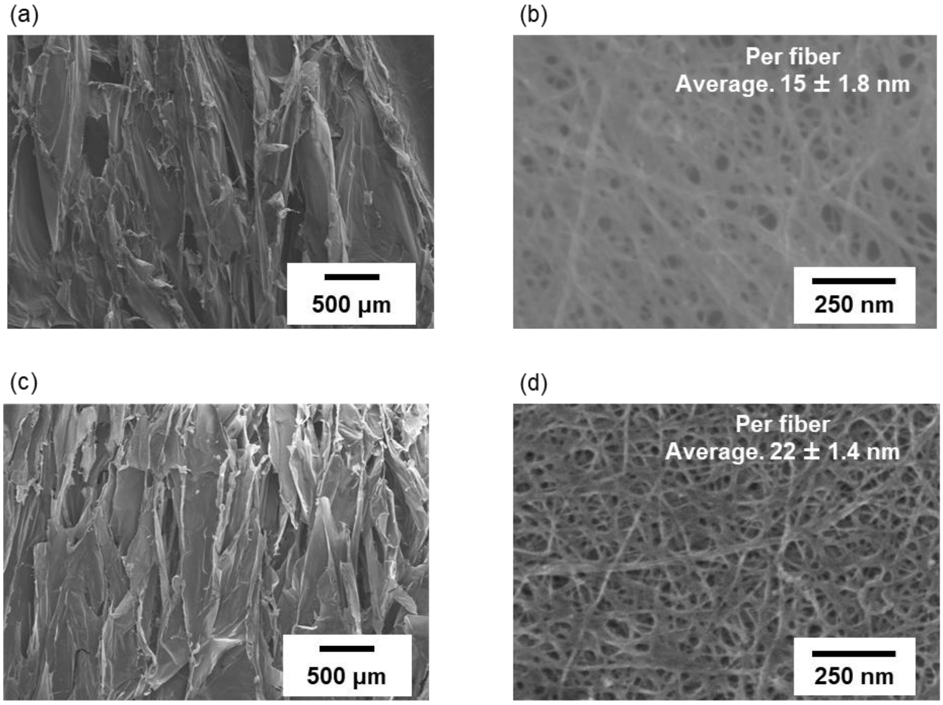

Figure S5. SEM images of the cross-sections ((**a**), (**c**)) and sheets surfaces ((**b**), (**d**)) of the CNT/CMC2(2.5_10) ((**a**), (**b**)) and CNT/CMC2(5_10) ((**c**), (**d**)).

Figure S6 shows the SEM images of the top parts of the CNT/CMC sheets and CNT/CMC sheet surfaces with different weight average molecular weights. Figures S6 (a), (c), and (e) correspond to CNT/CMC1(1.3_10) and (b), (d), and (f) to CNT/CMC3(1.3_10) sponges.

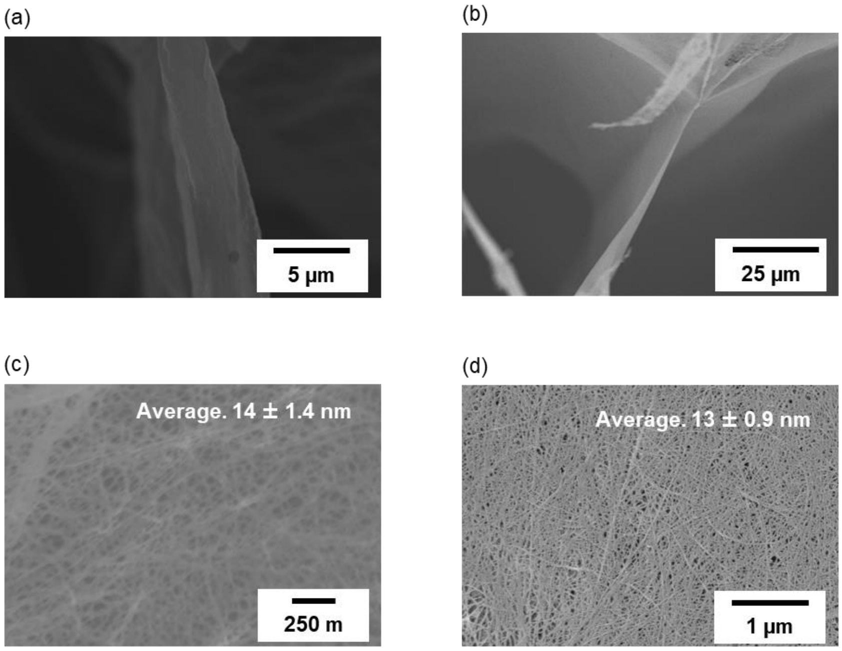

Figure S6. SEM images of the CNT/CMC sheets, and CNT/CMC sheet surfaces with different weight average molecular weight; (**a**) and (**c**) CNT/CMC1(1.3_10); (**b**) and (**d**) CNT/CMC3(1.3_10).

Figure S7 shows the stress-strain curves of CNT/CMC2 sponges with different densities at a CNT mass ratio of 10%. Figure S7 (a), (b), and (c) shows the stress-strain curves for the axial compression and Figure S7 (d), (e), and (f) shows the stress-strain curves for radial compression.

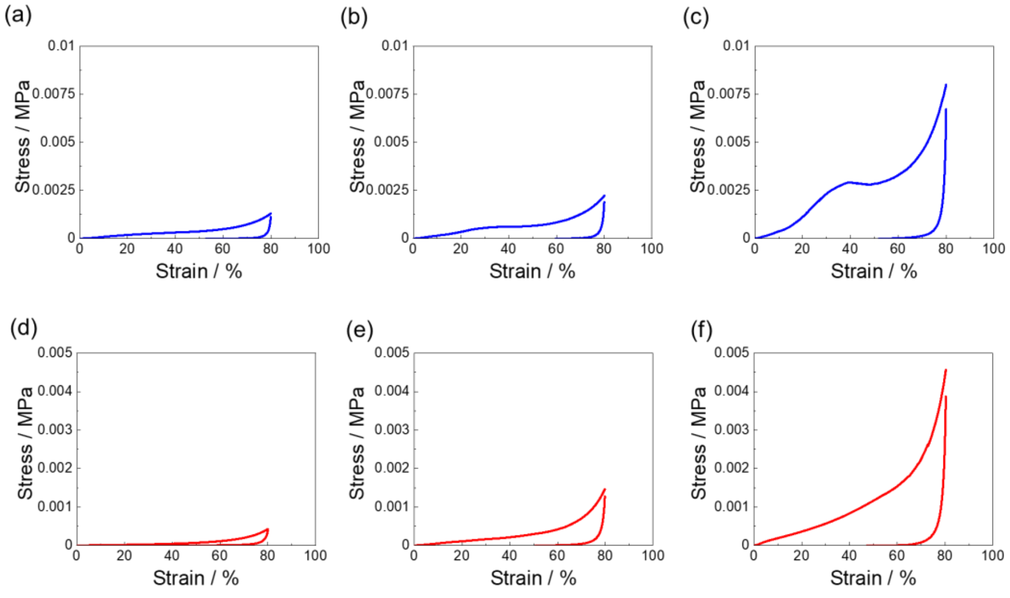

Figure S7. (**a**), (**b**), (**c**) Stress–strain curves for the axial compression; (**a**) CNT/CMC2 (1.3_10), (**b**) CNT/CMC2(2.5_10), (**c**) CNT/CMC2(5_10). (**d**), (**e**), (**f**) Stress–strain curves for radial compression; (**d**) CNT/CMC2(1.3_10), (**e**) CNT/CMC2(2.5_10), (**f**) CNT/CMC2(5_10).

Figure S8 shows Schematic diagrams of CNT/CMC sponge under compression. Figure S8 (a) shows CNT/CMC2(1.3_10) and (b) shows CNT/CMC2(5_10).

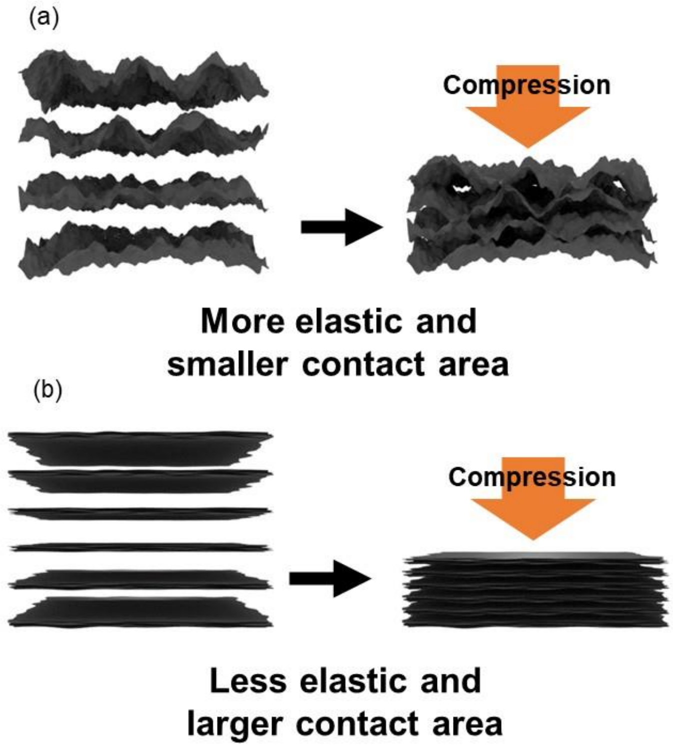

Figure S8. Schematic diagrams of CNT/CMC sponge under compression; (**a**) CNT/CMC2(1.3_10), (**b**) CNT/CMC2(5_10).

Figure S9 shows the relationship between CMC molecular weight and maximum stress when the CNT mass ratio was 10%.

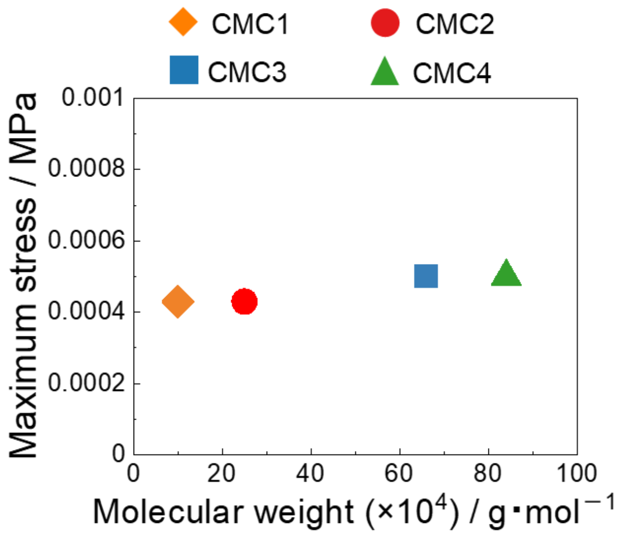

Figure S9. Relationship between CMC molecular weight and maximum stress when the CNT mass ratio was 10%.

Figure S10 shows the recovery rate during repeatedly compressing CNT/CMC1(1.3_10) at 80% strain. The 1st, 100th and, 1000th cycle showed recovery rate of 70%, 57% and 45%, respectively.

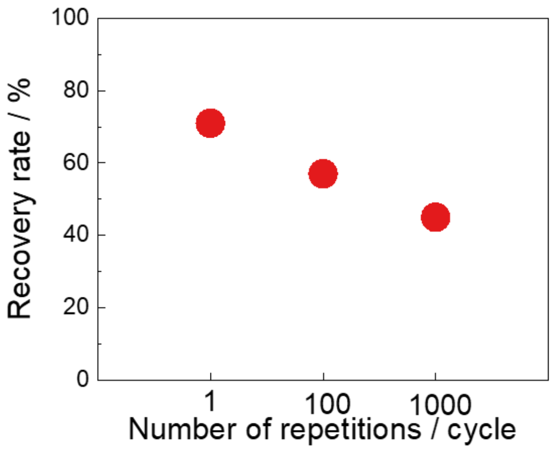

Figure S10. Relationship between cycle number and recovery rate when repeatedly compressing CNT/CMC1(1.3_10).

Table S2 shows pore volume and Brunauer-Emmett-Teller (BET) surface area of each CNT/CMC sponges obtained from BET N_2 sorption measurement.

Table S2. Pore volume and BET surface area obtained from BET N_2 sorption measurement.

Sample Name	Total Pore Volume(cm³/g) of Pores Less Than 40 nm in Diameter	BET Surface Area(m²/g)
CNT/CMC1(1.3_10)	0.022	17.6
CNT/CMC2(1.3_10)	0.086	30.0
CNT/CMC3(1.3_10)	0.025	16.5
CNT/CMC4(1.3_10)	0.029	16.5
CNT/CMC1(1.3_20)	0.178	54.4

Figure S11 shows the pore size distribution of the CNT/CMC sponges. Figure S11 shows (a) CNT/CMC1(1.3_10), (b) CNT/CMC2(1.3_10), (c) CNT/CMC3(1.3_10), (d) CNT/CMC4(1.3_10), and (e) CNT/CMC1(1.3_20).

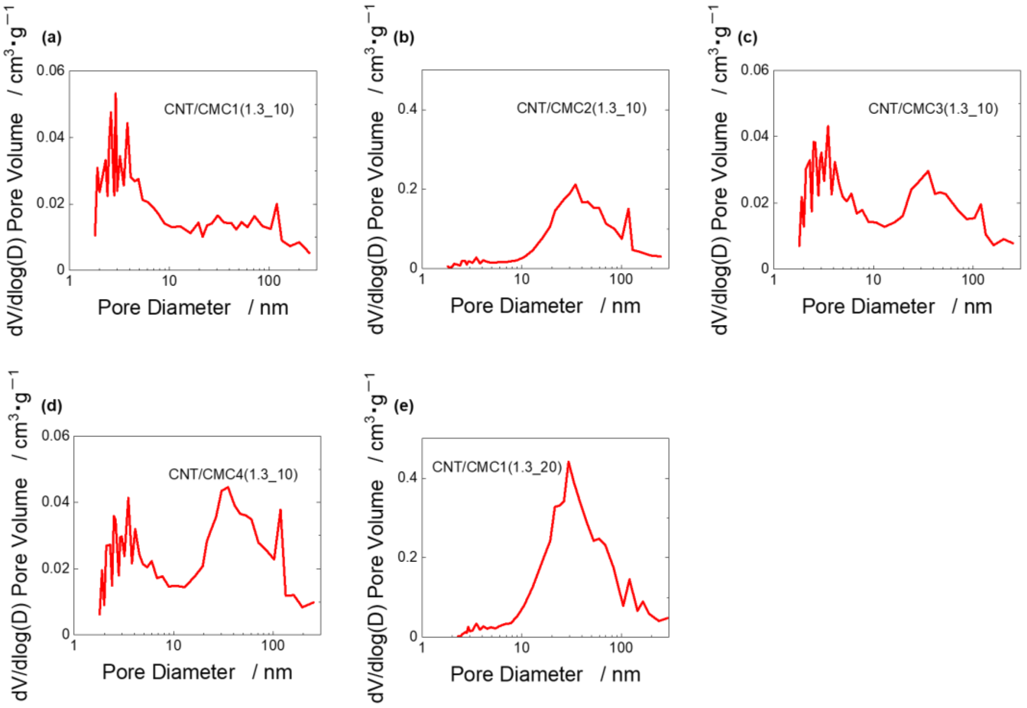

Figure S11. The pore-size distribution of the samples; (**a**): CNT/CMC1(1.3_10), (**b**): CNT/CMC2(1.3_10), (**c**): CNT/CMC3(1.3_10), (**d**): CNT/CMC4(1.3_10), (**e**): CNT/CMC1(1.3_20).

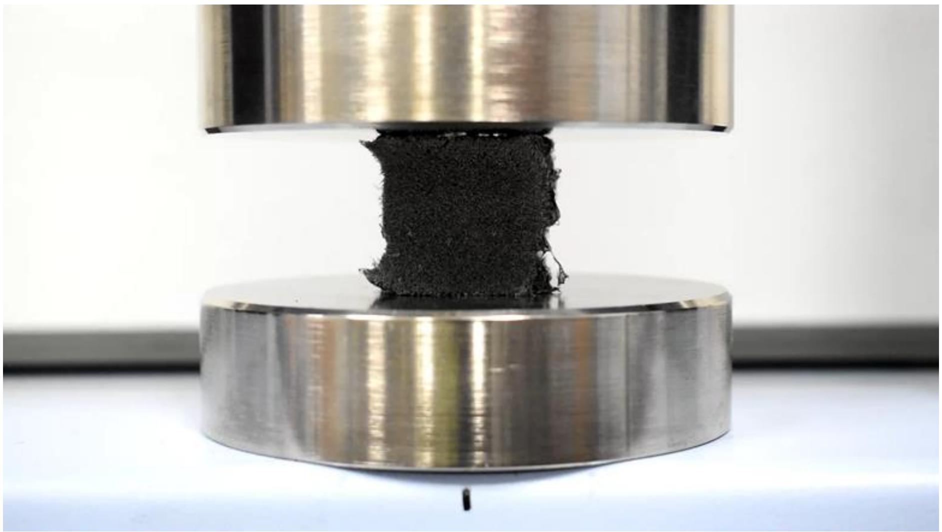

Video S1. supplementary movie of the compressed CNT/CMC1(1.3_10).
